# Complex Care Needs of People with Technology Dependence in Disaster Situations: A Scoping Review

**DOI:** 10.3390/healthcare13243305

**Published:** 2025-12-16

**Authors:** Anita Prasser, Joachim Beckert, Michael Köhler, Michael Ewers

**Affiliations:** Charité—Universitätsmedizin Berlin, Corporate Member of Freie Universität Berlin and Humboldt-Universität zu Berlin, Institute of Health and Nursing Science, Augustenburger Platz 1, 13353 Berlin, Germanymichael.ewers@charite.de (M.E.)

**Keywords:** disaster nursing, technology dependence, scoping review

## Abstract

**Background:** Providing complex care and support for people with technology dependence (PwTD) is challenging, even under routine conditions. During disasters, when health and power infrastructure are disrupted, the complex care of PwTD must be maintained under extreme conditions. This research aims to summarize the specific needs of PwTD in disasters and to describe how these needs are addressed in real-life events. **Methods:** We conducted a scoping review, searching four databases (CINAHL, MEDLINE, PsycInfo, SocINDEX) and the websites of relevant disaster relief organizations. A total of 43 of 2625 screened records were included. Content analysis was used to identify and cluster the needs of PwTD and the response to these needs. **Results:** Case reports were the most reported types of literature. It was repeatedly stated that PwTD have complex care needs that are often difficult to meet in disaster situations. The review identified three interdependent clusters of needs: clinical and supportive care needs, aids and supply needs, and access needs. The needs of patients and relatives were, as far as the situation allowed, met in accordance with existing plans and guidelines and, where these were found to be inadequate, through creative solutions devised by frontline nurses. **Conclusions:** We conclude that addressing the complex care needs of PwTD in disasters requires a strategy integrating structural preparedness, professional adaptability, and user participation. Nurses could play a key role in developing and implementing such strategies. This review provides a starting point to develop a more practice-oriented research agenda to achieve inclusive disaster risk management.

## 1. Introduction

People with technology dependence (PwTD) form a relatively small but diverse and challenging group of healthcare users. According to Brenner, et al. [1], technology dependence refers to “a wide range of clinical technology to support biological functioning across a dependency continuum, for a range of clinical conditions. They are initiated within a complex biopsychosocial context and have wide ranging sequelae for the child and family and health and social care delivery.” Other definitions, such as that of Spratling [2], highlight that “the terms technology and complex care should be included in the terminology and/or definition of all studies on children and adolescents who require technology”. Technology dependence can result from genetic and congenital disorders, complications related to prematurity and perinatal trauma, progressive illnesses like renal failure, neoplasia, and degenerative neurological conditions, as well as accidents [1]. Dependence can range from medical–technical support for respiration, cardiac function, nutrition, or dialysis to assistive devices for speech, vision, hearing, and mobility. The degree of dependence varies considerably and can extend from intermittent use of a single device to continuous dependence on multiple assistive devices over longer periods [1,2,3]. In general, PwTD rely on these technologies and assistive devices to sustain life and maintain independence.

Due to increased life expectancy and the growing availability and wider use of medical technology, the population of PwTD is growing [4]. Although the total number of this user group is difficult to determine and data on PwTD is not systematically recorded [5,6], trends in the literature suggest substantial growth [7,8]. For instance, an estimate of 2.6 million people worldwide received kidney replacement therapy in 2010, a number projected to increase to 5.4 million by 2030 due to population growth, improved survival of end-stage kidney disease, and better access to dialysis [8]. Overall trends show an estimation of 900 million people worldwide using assistive devices, excluding spectacles, with projections rising to 1.3 billion by 2050 [4].

PwTD have complex care needs encompassing clinical, psychosocial, and educational aspects. According to Spratling [2] complex care is a defining characteristic of the population of PwTD and “is associated with a high level of skilled nursing care”. Clinical needs include wound and line care, administration of medication (e.g., IV-therapy), suction management and oxygen therapy, clinical monitoring, and nutritional support [9]. Psychosocial needs concern the prevention or treatment of anxiety, depression, and post-traumatic stress disorder [1], as well as mitigating emotional and social burdens for family caregivers [10,11]. Education and information needs relate to managing medical technology [12], specific health conditions (e.g., renal failure), and self-management of care [13]. These overlapping needs illustrate the inherent complexity of care for PwTD, where clinical management, psychosocial stability, and patient education are tightly interrelated Comorbidities that are either related to the underlying cause of technology dependence or have other causes, as well as the variability and instability of the patients’ health and the associated uncertainty for all involved, add further complexity to the care situation [3,14].

Meeting these complex needs requires coordination across multiple care settings and professions. There are various professional groups that take on this coordination task, including doctors, paramedics, social workers and nurses [15]. The latter are considered particularly important because reliance on skilled nursing care is closely related to the complexity of care needs of PwTD [2]. Nurses are found across various health services. These services include all forms of home care such as home and community-based services (HCBS), hospital-at-home, and advanced home care, which allow PwTD to receive complex care in their own home [16]. Settings also include long-term care facilities and hospitals, as well as specialized care centers like dialysis centers or weaning facilities [13,16,17,18,19]. In all these settings, nurses from multiple specialties (e.g., nephrology, pneumology, neurology, intensive care) provide essential care, which may involve continuous monitoring depending on the severity of the health condition [2,20]. While PwTD need regular consultations with other healthcare professionals [9], such as specialized physicians, physical, occupational, and speech therapists, and social workers [21,22], nurses function as care coordinators or support other professions such as social workers in the interdisciplinary care coordination team, ensuring continuity of care for PwTD and supporting their families [15,23,24].

Even under everyday conditions, providing comprehensive care for PwTD places high demands on the care system and the service providers, which cannot always be met. As a result, PwTD often disproportionately experience access barriers and unmet needs compared to the general population [25]. Patient care is often inadequate and lacks a focus on results; safety issues are commonplace [20,26,27,28]. For example, PwTD often struggle to access nurses with specialized qualifications about their specific needs [29] and report availability and transportation barriers to healthcare [30,31]. Additionally, their families experience caregiver burden, especially when skilled care cannot be provided 24 h, PwTD living at home rely on family or informal caregivers [32].

These pre-existing challenges indicate that even under stable conditions, the care of PwTD is fragile, making it particularly vulnerable to disruption during disasters. As stated by the United Nations Office for Disaster Risk Reduction (UNDRR) a disaster constitutes a “serious disruption of the functioning of a community or a society at any scale due to hazardous events interacting with conditions of exposure, vulnerability and capacity, leading to one or more of the following: human, material, economic and environmental losses and impacts” [33]. They affect healthcare services and create cascading effects [34], including staff shortages when personnel are unable or unwilling to work [35], disruptions in power supply and medication or equipment supply chains [36,37], and facility evacuations or prolonged sheltering with limited resources [38,39]. Damaged or blocked roads can restrict access to care sites [40,41]. Some care sites may have been destroyed and others overwhelmed by patient surges [42]. Consequently, disasters pose particular challenges for care providers to maintain care for PwTD and adequately address their complex care needs under exceptional circumstances.

These challenges in providing continuous and needs-based care for PwTD during disasters have already been discussed in the literature [37,43,44,45]. Various guidelines state how this issue should be addressed [46,47,48,49,50] and what nursing competencies might be needed to maintain care during disasters [51]. However, only real-life disaster experiences can reveal which plans are actually feasible in practice and what other options are available to meet the complex needs of PwTD if the existing plans fall short. So far, no comprehensive overview exists outlining the evidence on specific care needs of PwTD during disasters, the resulting healthcare requirements arising from these needs, and how they are addressed under exceptional circumstances during real-life disaster conditions. Therefore, the aim of this study is twofold: (1) to identify the specific care needs of PwTD, the response to which is of existential importance to them, and (2) to describe how these specific care needs can be met even under exceptional circumstances. The results can inform more inclusive disaster management—taking into account the complex care needs of PwTD, supplement existing guidelines and disaster plans—and provide nurses with information on how to meet these specific needs creatively, even under extreme conditions.

## 2. Materials and Methods

### 2.1. Study Design

To achieve these aims, we used a scoping review methodology in accordance with the PRISMA-ScR guidelines and the methods recommended in the JBI Manual for Evidence Synthesis [52,53]. No protocol was preregistered due to the iterative and exploratory nature of this scoping review. Given the diversity of PwTD populations and disaster contexts, a scoping review is particularly suited to mapping the breadth of available knowledge and identify gaps.

### 2.2. Search Strategy

To identify peer-reviewed literature, we searched four relevant databases (MEDLINE, CINAHL, PsycInfo, SocINDEX) between November and December 2024. Terms commonly used to describe PwTD and their care in disasters were used to formulate search terms. For instance, as complex care for PwTD in literature is described to be closely related to skilled nursing care [2], this term was added to the concept of complex care needs. The search string was oriented on the PCC (population, concept, context) scheme [53] and combined Medical Subject Headings (MeSH 2022) with additional keywords in titles and abstracts (see Table 1 and Table A2). We adapted the search string for each database accordingly.

To capture relevant grey literature, we conducted a hand search in the reference lists of relevant publications as well as targeted searches on the websites of international disaster relief and humanitarian organizations such as the International Federation of Red Cross and Red Crescent Societies (IFRC), United Nations Office for Disaster Risk Reduction (UNDRR), and the Sphere Project for standards in humanitarian care [54] using terms from the original search string.

### 2.3. Eligibility Criteria

Publications were eligible if the full text was available in English or German and if they were either a peer-reviewed journal article, an empirical paper, or a report from a government agency or non-governmental organization (NGO). We restricted inclusion to publications after 2001 since the attention on the topic and research increased following the terror attacks on 11 September 2001.

We excluded publications if they were not set in a disaster context, did not address people with technology dependence, or did not provide information on the needs of PwTD. There were no restrictions on the quality of research. Editorials, blogs, letters, press releases, opinions, and commentaries, as well as systematic syntheses of evidence, including guidelines, were excluded as sources. However, the references of relevant systematic evidence syntheses were hand-searched for eligible studies.

### 2.4. Selection Process 

After duplicate removal, two independent reviewers (AP, JB) screened titles and abstracts against the predefined inclusion and exclusion criteria, using agreed operational definitions for key concepts (e.g., “people with technology dependence”, “needs”). Full texts of records meeting initial criteria were acquired for further screening for eligibility independently by two researchers (AP, JB) with a focus on needs and challenges in care provision in the context of disasters. Conflicts in the eligibility decisions were discussed and resolved by a third researcher (MK).

### 2.5. Data Extraction and Synthesis

Two researchers (AP, JB) independently extracted data using an iterative process, according to the recommendations of Pollock et al. [55], and organized the information in a data extraction matrix, which included author (s), study design, country, and information on the population, use of assistive technology, type of disasters, and specific needs. To better analyze in which conditions the needs of people with technology dependence are met in disasters, we extracted context factors (e.g., care setting, attending care workers).

We applied a basic content analysis [55] to synthesize the extracted information. A deductive–inductive approach was used, utilizing the data extraction matrix as an initial framework and open coding the needs and identifying overarching clusters.

## 3. Results

### 3.1. Study Selection

**Figure 1:**
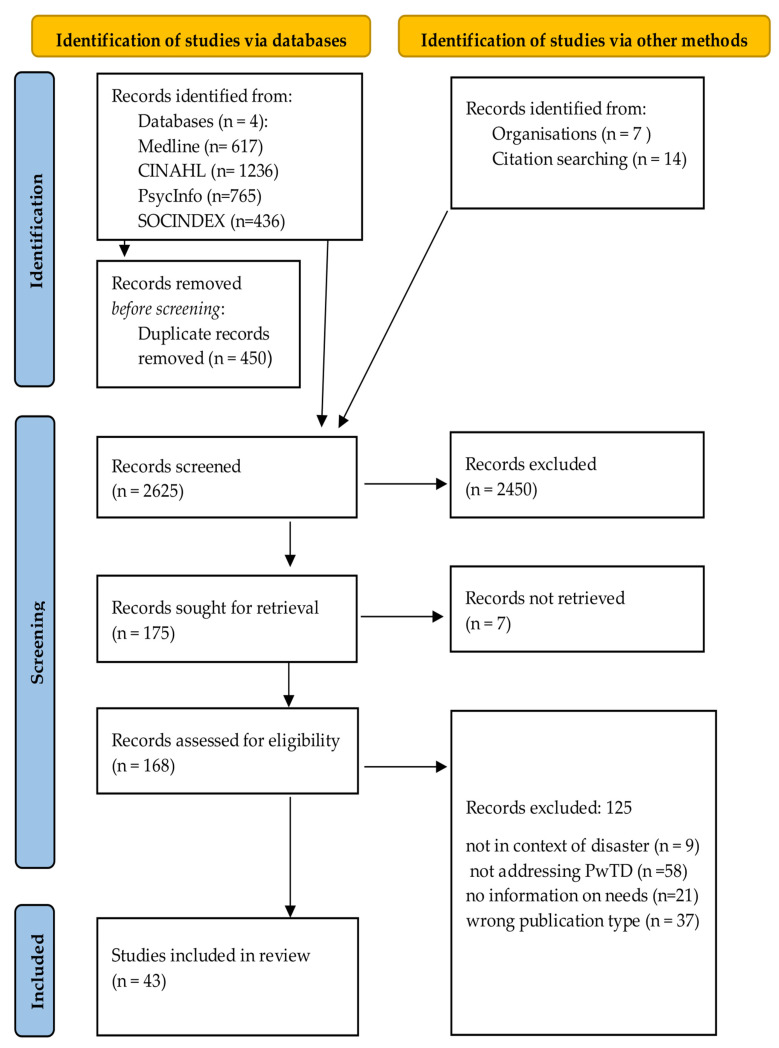
PRISMA flowchart for the selection of records.

Initially, the search yielded 3075 records. After removing duplicates and screening titles, keywords, and abstracts, 2450 records were excluded. Of the 168 full-text records retrieved, 125 were excluded for not meeting eligibility criteria. A total of 43 sources met the inclusion criteria, e.g., Table A1. The selection process is shown in Figure 1.

### 3.2. Identified Clusters of Needs of PwTDs and How They Were Addressed During Disasters

**Figure 2:**
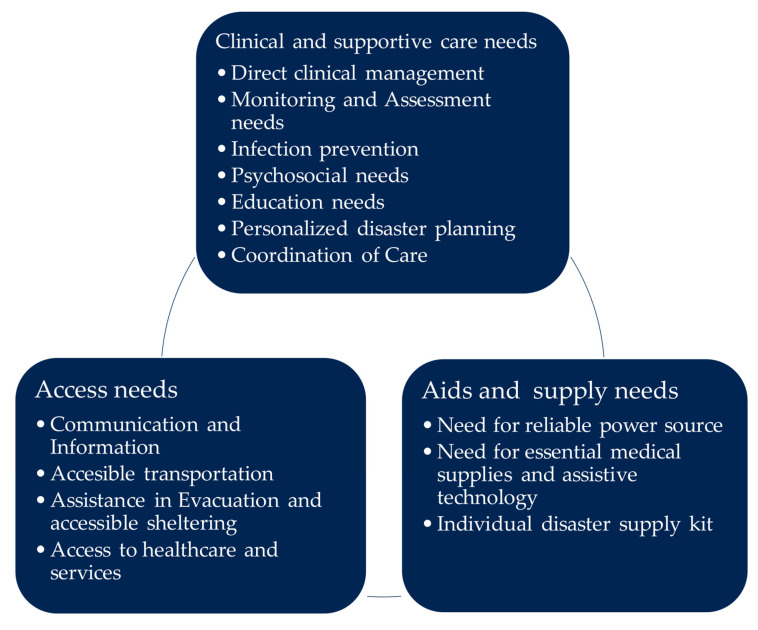
Clusters of the needs of PwTD in disasters.

Across the included studies, we identified three overarching clusters of care needs, the response to which is of existential importance for PwTD: “clinical and supportive care needs”, “aids and supply needs”, and “access needs”. Each cluster contained distinct needs, as illustrated in Figure 2. Within these clusters, we synthesized the needs described in each case and how these needs were specifically addressed during disasters based on empirical evidence.

#### 3.2.1. Clinical and Supportive Care Needs

##### Direct Clinical Management

Direct clinical management is a high priority for PwTD, encompassing strict medication, therapy, and dietary regimens [56,57,58,59,60,61] and routine clinical procedures like wound and line care, catheter management, and suction and oxygen management [62,63,64]. To address these clinical needs in disaster conditions, healthcare professionals were forced to adapt care provision to the available resources. These adaptations included changing continuous feeding with a pump to gravity flow or bolus feeding, maintaining ventilation with mechanical mask ventilation, or standardizing dialysis orders when individual prescriptions are unavailable [57,65]. For example, during hurricane Sandy, when specific formula was unavailable, a healthcare professional with expertise in enteral nutrition adapted an adult prescription using available infant nutrition and mixing instructions to meet adult calorie and protein requirements [66]. However, systemic challenges remained, as emergency food failed to meet dietary needs regarding dysphagia or other restrictions (specific nutrition formula and allergies) [56,61]. A lack of expertise and resources in disaster management organizations also compromised the safety of chronic wound care and other ongoing clinical interventions in the response and recovery phase [56,63,67].

##### Monitoring and Assessment Needs

Monitoring and assessment are essential for early detection of health deterioration of PwTD to enable immediate intervention, particularly given the instability of their health, regardless of whether they are in a care facility [57,68] or in a disaster shelter [69]. Disaster responses included initial screening by nurses to identify PwTD and assess their immediate care needs, health status, and functional ability [61,68]. In the case of particularly unstable PwTD, like preterm infants, expert monitoring of vital parameters by neonatal nurses was required, for example [57].

##### Infection Prevention

The crowded conditions in shelters combined with heightened susceptibility of PwTD and equipment contamination risks create a pressing need for infection prevention measures [70,71,72,73,74]. To respond to this need, measures included regular screening for infections, grouping symptomatic PwTD into cohorts [71,72,73,75,76], disinfecting assistive devices and contaminated equipment [65,74], and ensuring hygienic sheltering facilities [56].

##### Psychosocial Needs

PwTD have a psychosocial need to mitigate stress, fear, and anxiety in disasters, as all these symptoms are heightened due to the perceived threat during an event [77,78,79]. A viable response to this need identified in the literature was to arrange for the presence of familiar caregivers and licensed nurses in the various care settings and emergency shelters. These measures reportedly reduced anxiety and distress but also required a great deal of coordination [65,67,77].

##### Education Needs

PwTD and their families have information and education needs that are not always met by public disaster information [80]. These specific needs concern self-management and self-assessment of their health and the operation of assistive devices before, during, and after disasters [65,67,70,81]. In practice, the response to these information and education needs was often spontaneous, arising from the immediate challenges of the disaster context. Nurses and other providers were forced to provide targeted education in shelters and provisional care facilities, for instance, on non-dialytic management of end-stage kidney disease [73,75] and the use of alternative feeding methods (bolus, gravity bags) [66]. Educational topics also included the role of comorbidities in pandemics [71] and general disaster preparedness [65,82].

##### Personalized Disaster Planning

Personalized disaster and evacuation planning was recognized as a specific need for PwTD, as generic approaches are often insufficient for their individual requirements [57,59,83]. According to existing guidelines, individualized disaster plans should therefore be developed in anticipation of events with the support of nurses and other care providers [82,84]. However, evidence revealed gaps in such plans, often uncovered during real events or drills [85]. For instance, a cross-sectional study reported that PwTD and their families planned to go to the hospital in case of power failure but also reported that this plan was unlikely to work due to hospital lockdown and public transportation failure during an event [86].

##### Coordination of Care

Finally, the care provision attending to various needs must be coordinated to ensure safety and healthy recovery after the event. This includes coordinating essential services (e.g., dialysis) between shelters and alternative care facilities [87]. Evidence from case studies [60,75,87] and a prospective analysis [22] suggest that nurse-led coordination could be an effective response for improving care provision and recovery.

#### 3.2.2. Aids Supply Needs

##### Reliable Power Sources

PwTD depend on reliable, continuous power sources to operate their assistive technology—a need that becomes more critical during indefinite power outages common in disasters [37,60,63,65,81,86]. Documented solutions to meet this need include the provision of diesel generators in specialized facilities [65,75], backup batteries provided by facilities or stockpiled in emergency kits [57], and rapid repairs by technical personnel [62,75]. For instance, nurses delivered back-up batteries for people relying on home parenteral nutrition (HPN) when devices could not be charged during hurricane Sandy [66].

##### Essential Medical Supplies and Assistive Technology

In addition, PwTD need essential medical supplies and assistive technology, including clean water, consumable food (e.g., nutrition formula), medical oxygen [59,62,65,85,88], and medication and assistive device replacement [58,64]. This need is exacerbated by disaster-related logistical challenges, such as the short shelf life and refrigeration requirements of many supplies, and the difficulty of sterilizing equipment for reuse. In response, creative adaptations documented in the literature include healthcare providers supporting PwTD and their families to stockpile supplies, using simple glucose solutions as emergency sustenance, and even improvising with sterilization equipment from a tattoo parlor [59,77].

##### Individual Disaster Supply Kit

In order to compensate for the indefinite disruption of supply chains for specialized equipment and consumables, PwTD also need individualized disaster supply kits [57,65,80,85]. The reported response to this need was inconsistent. Nurses in some healthcare facilities had packed and distributed kits or backpacks tailored for individual PwTD [65,85]. However, a study interviewing PwTD living at home found that, apart from a stockpile of medication, no emergency kit was available for them [77]. Nurses compensated for the lack of individualized disaster kits by adapting care to standardized prescriptions [65] or using available substitutions fitting individual needs [59,66].

#### 3.2.3. Access Needs

Access to vital information and communication between providers, disaster management, and PwTD are essential. Adequate and accessible transportation, evacuation and sheltering, and access to essential healthcare services are also crucial in maintaining care for PwTD in disasters.

##### Communication and Information

Effective communication and access to reliable information are essential to maintain care for and independence of PwTD in disasters. PwTD need communication and information pathways to access care and make informed decisions during disasters [60,75,81,89,90]. These needs include maintaining communication with providers, receiving information on the availability of services [56,91], and overcoming disruptions to medical records and alarm systems that hinder care delivery [17,65,84]. In disaster response, emergency information cards were used containing information about medications, allergies, dialysis or nutrition prescriptions, emergency contacts, care providers, and medical history [58,59,60,75]. Physical records of care coordination also proved to be helpful in maintaining care when digital systems failed and data was lost [22].

##### Accessible Transportation

Beyond communication challenges, PwTD face access barriers related to transportation during disasters. Therefore, a critical need identified for PwTD during disasters is accessible transportation to reach shelters and alternate care sites [17,40,92] and for their home care nurses to reach them reliably. In disaster response, nurses adapted their schedules to account for long fuel lines and blocked roads, transported critical supplies themselves [66], and improvised transportation solutions, such as creating thermoregulated infant transport devices using blankets and plastic wrap material [57].

##### Evacuation and Accessible Sheltering

Additional access needs arise when PwTD have to evacuate or find appropriate shelter. PwTD need more time and assistance when forced to evacuate, and general population shelters often lack necessary accommodation for complex care needs [56,74,92,93,94]. Based on the literature, PwTD benefited from early evacuation due to their heightened vulnerability to service disruptions and power failures [40,75,84,94,95]. In disaster response, nurses and healthcare providers encouraged PwTD’s early evacuation and assisted them when care facilities were evacuated [57,66]. In some cases, specialized shelters for PwTD were established, providing reliable power, refrigeration, oxygen, and private care spaces and specialized nursing care [68,81,93]. Several sources highlight that these shelters were particularly useful when they took in not only PwTD but also their primary home care nurse or other familiar caregivers. This made it possible to maintain individualized care and relieve the burden on nurses responsible for the people in these shelters [56,68].

##### Access to Healthcare and Services

Finally, overall access to essential healthcare services might be compromised during disasters. Even in disaster situations, PwTD require continuity of care and unimpeded access to their essential healthcare contacts [70,79], including dialysis [60], home nursing [17,66], maintenance and repair of assistive devices [70,74], or emergency services if their condition deteriorates [37,90]. Their routine healthcare contacts might not be available during an event due to destroyed facilities, staff shortages, or face high patient surges [65]. Response strategies implemented by nurses included referring PwTD to outpatient dialysis facilities that operated during disasters [84], delivering care on adjusted schedules if they sheltered in place [66], or providing care in designated emergency shelters [67].

## 4. Discussion

This review set out to explore the specific care needs of PwTD in disasters and to describe how these needs can be met even under exceptional circumstances. The synthesis of the literature documents the range and complexity of these needs of PwTD, clustered in the areas of “clinical and supportive care”, “aids supply”, and “access to care”. The included sources mentioned numerous disaster scenarios in which formal guidelines and existing disaster plans for addressing these needs were inadequate or could only be implemented partially in practice. At the same time, however, they outlined how these challenges were overcome to meet the complex care needs of PwTD during disasters.

Developing disaster care guidelines and preparedness plans that are tailored for answering the complex care needs not only for PwTD but for the broad spectrum of so-called CMIST populations (communication, maintaining health, independence, support, safety and self-determination, transportation) with functional impairments and access problems [48,96] is of utmost importance. They are an essential first step towards a more inclusive disaster risk management and emergency response system, as has been called for by the United Nations and other international organizations for some time [50,97,98]. However, our analysis of the literature has shown that developing such inclusive disaster care guidelines and preparedness plans alone is insufficient. They must also be regularly tested, evaluated, and implemented in ways that reflect the realities of disasters. For instance, although existing guidelines propose measures like individualized stockpiles or emergency kits [47] and specialized shelter facilities for PwTD and others within a needs-based framework [48], the included studies indicate that these are only partially realized [85]. Evidence suggests that PwTD in home care settings tend to have less comprehensive stockpiles and supplies in alternate care sites do not match individual prescriptions [59,77]. Similarly, reports from real-life events point to challenges with screening and the provision of specialized sheltering, which can result in PwTD being housed in general shelters without appropriate facilities and personnel [56]. Although some countries have begun expanding civil protection and disaster control to individuals with complex care needs [48,81], their implementation and evaluation still seem to receive little attention. In addition, it would be appropriate to place greater emphasis on these guidelines and planning processes to ensure that people with complex problems are increasingly accommodated and cared for in community-based and home settings. As the literature shows, the prevailing conditions in these settings in the event of a disaster prove to be particularly challenging, which is why protecting those in need of complex care in their homes and communities deserves special attention in this endeavor.

When disaster response guidelines and emergency plans fell short and gaps in the care of PwTD emerged in disaster situations, these were often filled by spontaneous or improvised measures taken by qualified and experienced nurses [57,65]. For instance, nurses across settings used their expertise to improvise solutions when information or supplies were insufficient [66]. In other cases, nurses with clinical expertise were able to ensure patient safety even in unusual locations when their health deteriorated suddenly and evacuation was not possible, or no other care options were available [87]. This observed capacity for improvisation and immediate action appears to align with the core competencies of disaster nursing [51], emphasizing flexibility and decision making under constrained conditions. However, creativity must never go so far as to jeopardize the safety and health of the nurses themselves or the persons entrusted to their care. Differing from recommended care protocols should be a last resort and guided by ensuring quality of care and patient safety despite dire conditions. Frameworks regarding the standards of care in crisis and disasters could help to inform such efforts [99]. In order to identify risks for themselves or other helpers, the patients, and their relatives and to be able to control or avert these risks proactively, nurses require sound medical and nursing knowledge, sufficient clinical experience, and a high degree of professionalism [67]. This is even more important given that nurses in emergency and disaster care often have to respond spontaneously to unpredictable circumstances and act without being able to rely on the immediate support of colleagues or other clinical experts. The targeted deployment of skilled nurses in disaster risk management and emergency response appears to be one of the most promising measures for meeting the needs of people with complex needs during disasters and for maintaining their well-being even under difficult conditions, especially for PwTD.

The literature offers interesting insights into how providers respond to crises and disasters and how they sometimes deal with the challenges arising in exceptional situations in highly creative ways. However, the voices and perspectives of people with complex care needs and those in their social environment remain largely unconsidered. In many cases, the literature is characterized by a paternalistic attitude in which experts and other outsiders decide on patients’ needs and how to respond to them [49]. Examples of this are a primary focus on the technical aspect of care and vulnerabilities resulting from disruptions [62] or a focus on organizational challenges in disaster management [92]. The burden placed on family caregivers, especially in extreme situations, was also not addressed in the included literature. Even though, across the OECD countries, home care for complex care situations is promoted, it can lead to an increased reliance on family caregivers, especially when skilled care is not adequately funded [32]. Only a few studies focused on the lived experience of PwTD in disasters; these studies found that PwTD and their families considered their own disaster preparedness to be inadequate and were not familiar with guidelines from experts [77,80]. Sometimes they experienced problems during and after a disaster, they were not prepared for by their healthcare providers, like exacerbations arising from the access loss to regular dialysis and wound care [63]. It is extremely important to address this issue of user participation and actively involve people in emergency and disaster planning considerations. A sensitive, person-centered approach can help to alleviate irrational fears, enable needs-based precautions to be taken, and, above all, allow the specific experience of those most affected to be incorporated in the planning process. Often, PwTD and other patients with complex care needs have already gained experience with crises and exceptional situations during their long-term care. This could be put to work and actively taken up, for example, by the nurses who work with them to co-develop individual disaster plans. Including PwTD voices in preparedness planning and research could provide valuable insights into the feasibility of emergency and disaster response measures and, additionally, strengthen the person-centeredness of disaster preparedness and risk management.

### Limitations

While this study provides valuable insights, several limitations must be considered when interpreting the results.

First, the search strategy may have inadvertently missed relevant publications. While the terminology focused on PwTD and general descriptors of the population, it did not include specific diagnostic terms associated with technology dependence. As a result, studies focusing on particular diagnostic groups may have been overlooked.

Second, the found literature shows a dominance of reports regarding renal replacement therapy, leading to an underrepresentation of other PwTD such as children with special healthcare needs or those relying on enteral nutrition, oxygen therapy or mechanical ventilators, and their specific care needs in disasters.

Only publications in English and German language were included for pragmatic reasons. As a result, studies published in native languages, particularly those from the disaster-prone region of Asia, may have been overlooked. Additionally, the specifications of the search regarding nursing care may have led to underrepresentation of interdisciplinary care provided or care given by medical or public healthcare providers.

Third, the geographic distribution of included publications is skewed, with many of the studies originating from the United States. Therefore, the findings are influenced by the terminologies and frameworks used within the US context and may have limited transferability to other national contexts. For instance, as reported by the OECD [32], care systems differ greatly between nations regarding organization and financing systems. Some are tax-based, like the UK or Australia, while others have a health insurance-based system with a big private sector like the United States. Countries like Germany or Japan even have an insurance dedicated to long-term care [100]. In addition, there is a lack of insight into the extent to which measures have already been taken in other countries for inclusive disaster risk management for PwTD, what effects these measures are having, and what role nurses play in their implementation.

Finally, the nature of the evidence base presents a constraint. The body of literature is primarily composed of retrospective case studies and empirical analyses. While case studies can provide in-depth insights into specific contexts, the generalizability of their findings is limited. Similarly, the dominance of retrospective designs is a common challenge in disaster research due to the unpredictable nature of such events, which restricts the ability to collect data prospectively. The sole prospective study identified was incidentally affected by a disaster rather than designed for one, underscoring this methodological gap [22].

## 5. Conclusions

In order to be able to answer the complex care needs of PwTD and comparable patient groups during disasters, a complementary strategy that combines structural preparedness, professional adaptability, and user participation—including PwTD and their families—is required. Clinicians with dedicated disaster response roles within a multidisciplinary disaster management team and clinical expertise in emergency, critical care, or disaster nursing are a promising factor in the development, implementation, and evaluation of needs-based disaster response plans for people with complex care needs. As nurses play an important role in care delivery for PwTD across settings, their involvement can help mitigate care gaps for PwTD and prevent safety issues for these patients when existing plans fall short in the event of a disaster.

To further strengthen the evidence base, future studies should go beyond descriptive or retrospective designs and employ study designs that allow for both empirical depth and practical transferability. To create a more comprehensive body of research regarding the needs of PwTD in disasters, underrepresented subgroups (such as people relying on tube feeding, an allergy-specific diet, infusion therapy or respiration assistance) should be studied, and the benefits of multidisciplinary teams caring for people with complex needs in extreme conditions should be explored.

Future studies can build on the findings of this review to examine how the identified needs can be operationalized in disaster preparedness and response, particularly regarding the role of nurses within a multidisciplinary disaster management team, intersectoral and interdisciplinary cooperation (e.g., partnerships with home-care agencies, disaster management and utility companies), and organizational structures in different national contexts. Empirical studies extending this work could help to assess how far PwTD and their families are already integrated into disaster planning and what measures effectively sustain nursing care under disaster conditions. This review thus provides a foundation for a broader practice-oriented research agenda aimed at strengthening inclusive, needs-based, and person-centred disaster risk management.

## Figures and Tables

**Table 1:** Search strategy with keywords and MeSH terms for Medline.

Search Component	Keyword/(Title/Abstract)	MeSH-Term
Population	“medically fragile” “technology depend *” “technology assist *” “medically complex” “complex chronic health” “complex care need?” “special healthcare need?” “special need?” “at-risk” “CMIST”	Respiration artificial Renal dialysis Enteral nutrition Parenteral nutrition Heart-assistive devices Vulnerable populations Disabled persons
Concept	“functional need?” “techn * need?” “nursing care” “access”	Nursing care Nursing Health services needs and demand Health services for persons with disabilities Health resources Patient care
Context	“cris?s” “disaster?” “public health emergency”	Disasters

* wildcard operator to find all variations with different endings.

## Data Availability

No new data were created or analyzed in this study.

## Abbreviations

The following abbreviations are used in this manuscript:

PwTD	People with technology dependence
MeSH	Medical subject headings
IFRC	International Federation of Red Cross and Red Crescent Societies
UNDRR	United Nations Office for Disaster Risk Reduction
CMIST	Communication, maintaining health, independence, support, safety and self-determination, transportation

## Appendix A

**Table A1:** Overview of the included studies.

Author, Year Country	Aim and Study Design	Population
Al Amin, Morrison, Kabir and Sajib [71], Bangladesh	Short report using routine data summarizing strategies to mitigate the sufferings of non-COVID patients with chronic kidney disease.	People suffering from chronic kidney disease using dialysis during the COVID-19 pandemic
Arichi, Cadwgan, McDonald, Patel, Turner, Barkey, Lumsden and Fairhurst [79], USA	Cross-sectional survey highlighting the impact of the initial pandemic response on health, education, and social care for children and young people with severe physical neurodisability and multiple comorbidities.	Children or young people with severe neurodisabilities
Baker and Baker [80], USA	Cross-sectional baseline study within a larger longitudinal study to explore disaster preparedness levels among families with children with special healthcare needs (CSHCN) and educate on preparedness for this population.	Family caregivers of children with special healthcare needs
Baker, Baker and Flagg [82], USA	Quasi-experimental pretest/posttest intervention study to determine the short-term effectiveness of a brief patient education intervention in increasing disaster preparedness among families of CSHCN.	Family caregivers of children with special healthcare needs
Benigno, Kleinitz, Calina, Alcido, Gohy and Hall [89],Philippines	Report outlining needs assessments and activities conducted by WHO and other specialists to improve access to rehabilitation and services for people with disabilities and injuries after the Haiyan typhoon.	People with disabilities relying on various assistive technology.
Berry, Soltau, Richmond, Kieltyka, Tran and Williams [22], USA	Prospective study to evaluate the ability of nurse care coordinators to enhance a pediatric clinic’s ability to meet medical home objectives and improve service receipt for families of children with special healthcare needs before and after a hurricane.	Family caregivers of children with special healthcare needs
Cary and Schroeder [65], USA	Case study on the lessons learned from caring for patients on kidney dialysis in a disaster in the renal care group in Baton Rouge during Hurricane Katrina.	Nurses caring for patients receiving hemodialysis
Davis, O’Shea and Beach [62], USA	Hazard vulnerability analysis exploring the risks and specific needs that VAD patients could face in a disaster situation.	People with ventricular assist devices
Deal, Fountain, Russell-Broaddus and Stanley-Hermanns [67], USA	Case study discussing challenges and opportunities encountered by nurses volunteering in special-needs shelters.	Nurses caring for people in special-needs shelters
Fannin, Brannen, Howell and Martin [69], USA	Quasi-experimental study evaluating Medical Reserve Corps volunteers and public health workers in conducting chronic care triage by use of a rubric prior to sheltering to connect survivors with services.	Medical Reserve Corps volunteers, public health workers, and personal care assistants
Finkelstein and Finkelstein [77], Israel	Qualitative interview aimed at understanding how people with disabilities perceive emergencies and their needs in preparing for such situations.	People with disabilities using wheelchairs and ventilators
Gershon, Muska, Zhi and Kraus [92], USA	Cross-sectional study assessing disaster planning of local offices of emergency management with respect to people with disabilities.	Local disaster planning offices planning for people with disabilities using mobility and respiratory assistive devices
Goodhue, Demeter, Burke, Toor, Upperman and Merritt [59], USA	Mixed-methods pilot study evaluating disaster preparedness of families with children dependent on enteral or parenteral nutrition.	Families of children relying on EN/PN
Hoffman, Fagan, Casas-Melley, Wei and Hebra [68], USA	Case study on the measures taken to protect children with special healthcare needs during hurricane Irma in a children’s hospital in Florida.	Children with special healthcare needs using ventilators and other assistive technologies
Holmes, Williams, Wong, Smith, Bandzuh and Uejio [56], USA	Case study presenting an evaluation framework to assess factors influencing the emergency response and management of the Special Needs Shelter Program in Monroe County, Florida during Hurricane Irma in 2017.	People in a special needs shelter using various assistive devices
Kelman, Finne, Bogdanov, Worrall, Margolis, Rising, MaCurdy and Lurie [90], USA	Retrospective cohort study using Medicare/Medicaid claims data to analyze care patterns and mortality of kidney disease patients in areas most affected by Hurricane Sandy, aiming to inform disaster preparedness and mitigate adverse outcomes for this population.	People with end-stage renal disease (ESRD) relying on dialysis
Khorram-Manesh, Yttermyr, Sörensson and Carlström [17], Sweden	Cross-sectional study assessing the overall risks that influence advanced care at home patients in Sweden.	People receiving advanced care at home
Kleinpeter [91], USA	Case study reviewing ESRD patients and services before and after Hurricanes Katrina and Rita, covering disaster preparation, recovery, mitigation activities, and health policy recommendations for patients, healthcare professionals, and services relevant to other natural disasters.	People with ESRD relying on dialysis
Kolwaite, Hlady, Simon, Cadwell, Daley, Fleischauer, May and Thoroughman [88], USA	Cross-sectional study conducted through community assessment for public health emergency response following severe weather events, assessing household health status, preparedness, and shelter needs for future planning.	Randomly selected households in Pike Country, Kentucky
Kopp, Ball, Cohen, Kenney, Lempert, Miller, Muntner, Qureshi and Yelton [84], USA	Retrospective Case report of disaster response following the 2005 hurricane season and the Kashmir earthquake, assessing the severe impact on dialysis patient care and facility operations.	People with ESRD relying on dialysis
Koyama, Fuse, Hagiwara, Matsumoto, Shiraishi, Masuno, Miyauchi, Kawai and Yokota [40], Japan	Case study describing difficulties in delivering medical supplies, providing medical care and evacuating patients in a hospital after the earthquake and nuclear disaster in Fukushima.	Hospital staff and patients relying on ventilators or dialysis
Löfqvist, Oskarsson, Brändström, Vuorio and Haney [83], Sweden	Cross sectional study assessing ICU evacuation preparedness for fire in all hospitals within Sweden’s national public healthcare system.	Staff and patients in intensive care units in Sweden
Lukowsky, Dobalian, Goldfarb, Kalantar-Zadeh and Der-Martirosian [72], USA	Retrospective longitudinal cohort study examining dialysis and healthcare service utilization by ESRD patients in Manhattan receiving hemodialysis pre- and post- Hurricane Sandy.	Patients with ESRD relying on dialysis
Ma, Cohen and Lee [85], USA	Qualitative interview study exploring how NICU personnel in California hospitals managed neonatal transfers during recent wildfires, with the goal of sharing lessons learned to enhance disaster preparedness for healthcare teams.	Staff and pediatric patients in a neonatal intensive care unit during wildfires
McKinney, McKinney and Swartz [78], South Africa	Case study exploring challenges people with disabilities experience accessing healthcare facilities during the COVID-19 pandemic.	People with disabilities relying on wheelchairs or/and ventilators
Mellgard, Abramson, Okamura and Weerahandi [63], USA	Case study exploring Hurricane Marias impact on a resident of Puerto Rico with chronic health problems seeking healthcare in New York.	Person with multiple chronic conditions relying on dialysis
Molinari, Chen, Krishna and Morris [37], USA	Secondary data analysis focused on estimating the national prevalence of individuals relying on electricity-dependent technology at home and who are at risk during a power outage.	People depending on electrically powered technology
Motoki, Mori, Kaji, Nonami, Fukano, Kayano, Kawada, Kimura, Yasui, Ueki and Ugai [70], Japan	Qualitative interview study aimed at developing pamphlets to help patients with diabetes, rheumatic diseases, and chronic respiratory disease, and those undergoing dialysis, to manage their conditions during disasters.	People suffering from diabetes, rheumatic disease, chronic respiratory disease or needing dialysis and being affected by an Earthquake or typhoon
Naghavi, Faramarzi, Abbasi and Badakhshiyan [74], Iran	Qualitative interview study explored the challenges Iranian assistive technology users faced in accessing health information and care during the COVID-19 pandemic.	People using assistive devices
Nakayama, Tanaka, Uematsu, Kikuchi, Hino-Fukuyo, Morimoto, Sakamoto, Tsuchiya and Kure [81], Japan	Case study examining the impact of the 2011 Eastern Japan earthquake and subsequent blackout on pediatric patients using medical devices.	Children using assistive technology
Orlando, Bernard and Mathews [57], USA	Case study exploring nursing care challenges and lessons from neonatal units in New Orleans during and after Hurricane Katrina, offering guidance to support disaster education for neonatal nurses.	Staff and Patients in NICUs
Rauch, Baumberger, Moise, von Elm and Reinhardt [64], Haiti	Pilot study using an ICF-based tool to assess rehabilitation needs in person with spinal cord injuries following the 2010 earthquake in Haiti.	People with spinal cord injuries needing assistive technology
Sakashita, Matthews and Yamamoto [86], USA	Cross-sectional survey of caregivers for children with technology dependence with special healthcare needs, assessing household preparedness for electrical power failures, device backup power availability, and emergency contingency planning.	Children with special healthcare needs
Sekkarie, Zanabli, Rifai, Murad and Al-Makki [73], Syria	Case study on the impact of the conflict in Syria on the hemodialysis system and ESRD patients focusing on the collapsed healthcare in Aleppo, Idlib and Homs.	Patients with end-stage renal disease (ESRD)
Shakour, Mithani, Kopp, Shepherd, Nogueira, Espinel and Shultz [75], USA	Case study on lessons learned from dialysis providers disaster response in providing patient support during Hurricane Ian in Florida.	People with ESRD relying on dialysis
Shapira, Aharonson-Daniel, Clarfield and Feder-Bubis [58], Israel	Mixed-methods study exploring the perceptions and needs of community-dwelling medically vulnerable individuals in Israel to inform future preparedness efforts.	Medically vulnerable relying on dialysis and other technologies
Skewes [87], Australia	Case study reporting on the challenges in maintaining renal dialysis services during the Toowoomba flood disaster.	Staff and patients of a dialysis facility
Tashiro, Kawakami, Oka, Liu, Nishimura, Ogawa, Hagai, Yamamoto, Yazawa and Liu [61], Japan	Cross-sectional study estimating the number of community-dwelling elderly care recipients who require specific food preparations.	Community-dwelling elderly recipients of long-term care
Tatsuki [93], Japan	Case study exploring challenges for people with functional needs following the 2011 Great East Japan Earthquake.	People with functional needs
Trento and Allen [66], USA	Retrospective case report analyzing the impact of Hurricane Sandy on home parenteral and enteral nutrition consumers.	People relying on EN/PN
Tuglular, Luyckx, Vanholder, Skoberne, Wiecek, Nistor, Pawlowicz-Szlarska, Shroff, Ivanov, Eckardt, Noruisiene, Gallego, Loboda and Sever [60], Ukraine	Case study summarizing major challenges and lessons learned by the renal disaster task force during the conflict in Ukraine.	People with ESRD relying on dialysis
United Nations Office for Disaster Risk Reduction [94], Switzerland	Report assessing disaster preparedness, evacuation and assistance planning for people with disabilities and to evaluate the accessibility of early warning and risk information and determining if disaster risk reduction plans include their specific needs.	People with disabilities using various assistive technology
United Nations Office for Disaster Risk Reduction [95], Switzerland	Scoping report exploring how linking disaster risk reduction (DRR) with the Convention on the Rights of Persons with Disabilities (CRPD) can strengthen protections for people with disabilities in disasters.	People with disabilities using various assistive technology

**Table A2:** Complete Search String for MEDLINE.

Search	Search Term
1.	“medically fragile”.ab,ti.
2.	“technology depend *”.ab,ti.
3.	“technology assist *”.ab,ti.
4.	“medically complex”.ab,ti.
5.	“complex chronic health”.ab,ti.
6.	“complex care need?”.ab,ti.
7.	“special healthcare need?”.ab,ti.
8.	“special need?”.ab,ti.
9.	“at-risk”.ab,ti.
10.	CMIST.ab,ti.
11.	Respiration, Artificial/
12.	Renal Dialysis/
13.	Enteral Nutrition/
14.	Parenteral Nutrition/
15.	Heart-Assist Devices/
16.	Vulnerable Populations/
17.	Disabled Persons/
18.	Nursing Care/
19.	Nursing/
20.	“Health Services Needs and Demand”/
21.	“Health Services for Persons with Disabilities”/
22.	Health Resources/
23.	Patient Care/
24.	“functional need?”.ab,ti.
25.	“techn * need?”.ab,ti.
26.	“nursing care”.ab,ti.
27.	“access”.ab,ti.
28.	Disasters/
29.	“cris?s”.ab,ti.
30.	disaster?.ab,ti.
31.	“public health emergency”.ab,ti.
32.	1. OR 2. OR 3. OR 4. OR 5. OR 6. OR 7. OR 8. OR 9. OR 10. OR 11. OR 12. OR 13. OR14. OR 15. OR 16. OR 17.
33.	18. OR 19. OR 20. OR 21. OR 22. OR 23 OR 24. OR 25. OR 26. OR 27.
34.	28. OR 29. OR 30. OR 31.
35.	32. AND 33. AND 34.
36.	Limit 35 to year 2001—current
37.	Limit 36 to language: English, German

* wildcard operator to find all variations with different endings.
